# Mediterranean spotted fever in Spain, 1997-2014: Epidemiological situation based on hospitalization records

**DOI:** 10.1371/journal.pone.0174745

**Published:** 2017-03-29

**Authors:** Zaida Herrador, Amalia Fernandez-Martinez, Diana Gomez-Barroso, Inmaculada León, Carmen Vieira, Antonio Muro, Agustín Benito

**Affiliations:** 1 National Centre for Tropical Medicine, Health Institute Carlos III (ISCIII in Spanish), Madrid, Spain; 2 Network Biomedical Research on Tropical Diseases (RICET in Spanish), Madrid, Spain; 3 Network Biomedical Research Centre in Epidemiology and Public Health (CIBERESP in Spanish), Madrid, Spain; 4 National Centre of Epidemiology, Health Institute Carlos III (ISCIII in Spanish), Madrid, Spain; 5 Unidad de Investigación Enfermedades Infecciosas y Tropicales (e-INTRO), Instituto de Investigación Biomédica de Salamanca-Centro de Investigación de Enfermedades Tropicales de la Universidad de Salamanca (IBSAL-CIETUS), Facultad de Farmacia, Universidad de Salamanca, Salamanca, Spain; University of Minnesota, UNITED STATES

## Abstract

**Introduction:**

Mediterranean spotted fever (MSF) is a zoonotic disease caused by *Rickettsia conorii*. In Spain, deficiencies in the official reporting result in misreporting of this disease. This study aims to describe the clinical and temporal-spatial characteristics of MSF hospitalizations between 1997 and 2014.

**Materials and methods:**

We performed a retrospective descriptive study using the Hospitalization Minimum Data Set (CMBD). All CMBD’s hospital discharges with ICD-9 CM code 082.1 were analyzed. Hospitalization rates were calculated and clinical characteristics were described. Spatial distribution of cases and their temporal behavior were also assessed.

**Results:**

A total of 4,735 hospitalizations with MSF diagnosis were recorded during the study period, out of which 62.2% were male, mean age of 48. Diabetes mellitus, alcohol dependence syndrome, and chronic liver disease occurred in 10.8%, 2.4% and 2.8% hospitalizations, respectively. The median annual hospitalization rate showed a decreasing trend from a maximum of 12.9 in 1997 to a minimum rate of 3.1 in 2014. Most admissions occurred during the summer, showing a significant annual seasonal behavior. Important regional differences were found.

**Discussion:**

Although MSF hospitalization rates have decreased considerably, it remains a public health problem due to its severity and economic impact. Therefore, it would be desirable to improve its oversight and surveillance.

## Background

Mediterranean spotted fever (MSF), or ‘boutonneuse’ fever, is caused by *Rickettsia conorii* and is transmitted by the brown dog tick *Rhipicephalus sanguineus*. This tick is relatively host-specific, and rarely feeds on people unless its preferred host—the dog—is not available [1]. It was first described in 1910 as a disease that caused high fever and spots [2]. In Europe, it is endemic in the Mediterranean area, where most cases are encountered in the summer when the tick vectors are highly active. In Southern Europe many cases can also occurred in spring, especially if this season is particularly warm and, thus the abundance and activity of these ticks is high [3].

MSF is characterized, as other rickettsioses, by fever, headaches, and maculopapular rash. The tick bite causes a characteristic rash and a distinct mark—namely, a tache noire (black spot) at the site of the bite. Flu-like symptoms are also common. Eschars are rarely multiple. In some patients, the eruption is papulovesicular; this form is more common in adults in Africa. In other patients, the only symptom is an isolated lymphadenopathy [2,3].

MSF is usually a mild disease, but severe complications including hepatic, renal, cardiac, neurological and multiorgan involvement can occur in about 6–10% of cases, often resulting from delayed diagnosis [4]. Severe MSF can also be accompanied by clinical features that are more frequently observed in immunocompromised patients. The pathogenesis of MSF complications results from vascular injury, which may be responsible for organ dysfunction of different organs [5]. The mortality rate is usually estimated at around 2.5% [2,3].

Mild forms of the disease are usually observed in children, while complications of MSF are more common in patients with underlying disease or in elderly persons [3]. Nevertheless, in recent times an increasing number of complicated MSF cases are being reported, even in the absence of predisposing conditions [4]. Classic human risk factors for severe MSF include elderly people, cirrhosis and chronic alcoholism [6,7]. Other risk factors include the following: immunocompromised situations, glucose-6-phosphate dehydrogenase (G6PD) deficiency and diabetes [2,8]. On the other hand, the risk of ticks transmitting *R*. *conorii* and, consequently, the incidence of MSF are also dependent on several parameters [6]: (a) the prevalence of rickettsia-infected ticks, which can vary greatly; (b) the affinity of a specific tick for humans and/or (c) the abundance of the tick itself, which is influenced by many factors, including climatic and ecological conditions, [9]. There is no vaccine available for MSF and the only preventive measure is the surveillance of attached ticks after exposure, apart from the use of repellents [10].

MSF was first described in Tunisia and was soon reported in other regions around the Mediterranean basin, including northern Africa and southern Europe. In the last decade, the use of molecular biology techniques and the identification of species have enable to better define the epidemiological setting of the disease and to correlate more severe clinical forms with new Rickettsia’s species [2]. In Spain, MSF is endemic. The dog is the main domestic reservoir, although wild mammals have also been found infected with *R*. *conorii* [11]. Occasionally, outbreaks might occur [12]. Few sero-epidemiological population-based studies of *Rickettsia* spp. infections have been carried out in different regions of Spain, finding specific antibodies to *R*. *conorii* in 9% of inhabitants in Catalonia and 5% of inhabitants in Madrid [10,13]. However, the real incidence of MSF remains unknown. Notification of MSF was mandatory only in some regions until 2015, when the reporting became mandatory at national level [14]. Moreover, there is no national control programme against MSF [10,12]. In the present study MSF related hospitalizations in Spain from 1997 to 2014, were analyzed in terms of time, geographical distribution, and disease related individual characteristics in an attempt to determine epidemiologic picture of the disease. In addition, MSF reported cases to official authorities were also assessed in order to evaluate and compare the information provided by these two databases.

## Methods

### Ethics statement

The study involves the use of patient medical data from The Spanish Centralized Hospital Discharge Database (CMBD). Data are hosted by the Ministry of Health Social Services and Equality (MSSSI). Researchers working in public and private institutions can request the databases by filling, signing and sending a questionnaire available at the MSSSI website. In this questionnaire a signed Confidentiality Commitment is required. All data are anonymized and de-identified by the MSSSI before it is provided to applicants. According to this Confidentiality Commitment signed with the MSSSI, researchers cannot provide the data to other researchers that must request the data directly to the MSSSI [15].

### Study and data analysis

We performed a retrospective descriptive study using the Centralized Hospital Discharge Database (CMBD in Spanish) from January 1st, 1997 to December 31st, 2014. International Classification of Diseases, Ninth Revision, Clinical Modification (ICD 9 CM), the ICD version employed during the study period, was used for this purpose [16]. Registers with ICD-9 CM code 082.1 (“boutonneuse fever”) placed in any diagnostic position were analyzed.

The CMBD database receives notification from around 98% of the public hospitals in Spain [15]. The National Health System (NHS) provides free medical care to 99.5% of the Spanish population, although those persons not covered by the NHS can be attended at the public hospitals. Private hospitals represent only a small proportion of all hospital admissions. Since 2005, CMBD also has a gradual coverage from private hospitals [17].

For each entry, we collected socio-demographic (sex, age and autonomous community of residency) and clinical data (other diagnosis, type and department of admission, average length of hospitalization, non-invasive procedures and history of surgical intervention during the hospitalization, re-admission and outcome.

The average number of hospitalizations per year, autonomous community (CCAA in Spanish) and province were calculated in order to assess temporal and geographical patterns. Official population figures of the Spanish regions and provinces were used as population at risk for the study period 1998–2014 [18]. Data was missing for 1997, thus the population data from the Intercensus Population Estimates were used for that year [19]. Comparison of the CMBD data and the records from the National Epidemiological Surveillance Network (RENAVE in Spanish) database from 2005 to 2014 was also carried out.

The temporal behavior of the hospitalizations due to MSF was assessed using the classical approach to time series analysis. Linear regression was used to assess the trend, and the periodogram of the time serie was computed to study its seasonality. Finally, we performed a Poisson regression analysis with the monthly number of MSF hospitalizations as dependent variable, including trend and seasonality as independent variables by using the sine and cosine functions.

The hospitalizations rates by provinces were mapped using the Geographical Information System Arcgis version 10.0. Data analysis was performed using STATA software version 14.

## Results

### Sociodemographic and clinical characteristics of MSF hospitalizations

Between 1997 and 2014, 4,735 hospitalizations with diagnosis of MSF in any diagnostic position were recorded in the CMBD database. A total of 62.2% hospitalized were male, predominating in all age-groups. The mean age was 48 years (range 0–95) with the 46–64 and >=65 age groups being slightly more represented. The predominant admission type was “urgent” (96.7%). The 70.3% were hospitalized for 7 or less days. The 97.8% of hospitalizations were discharged at home, death occurring only in 0.8%. Only 98/4,735 (2.1%) were re-admissions(Table 1).

**Table 1:** Clinical characteristics of Mediterranean spotted fever hospitalizations (n = 4,735), 1997–2014, Spain.

Variables	n (%)
**Sex**	
Male	2945 (62.2)
Female	1790 (37.8)
**Age-groups**	
<15 y	731 (15.4)
16–44 y	1188 (25.1)
45–64 y	1383 (29.2)
>=65 y	1433 (30.3)
**Type of admission**	
Urgent	4577 (96.7)
Programmed	154 (3.3)
Others/unknown	4 (0.1)
**Surgical intervention**	
No	4453 (94.0)
Yes	282 (6.0)
**Lenght of stay**	
≤ 7 days	3331 (70.3)
> 7 days	1404 (29.7)
**Type of discharge**	
Home	4,630 (97.8)
Transfer	29 (0.6)
Others/unknown	38 (0.8)
Exitus	38 (0.8)
**Readmission**	
No	4,637 (97.9)
Yes	98 (2.1)

For all hospitalizations with MSF diagnosis in any diagnostic position, the associated diagnoses commonly considered in the literature as risk factors [2, 18] were investigated. Diabetes mellitus was found in 512 hospitalization records out of 4.735 (10.8%), alcohol dependence syndrome occurred in 113 (2.4%) and chronic liver disease and cirrhosis occurred in 132 hospitalizations (2.8%) including alcoholic cirrhosis of liver (0.3%), cirrhosis of liver without mention of alcohol (0.2%) and biliary cirrhosis (0.02%). There are 38 hospitalizations diagnosed as seropositive for HIV (0.8%) (Table 2).

**Table 2:** Selected co-diagnoses of Mediterranean spotted fever placed in any diagnostic position, CMBD database, 1997–2014, Spain.

ICD-9 CM groups	Co-diagnoses	ICD-9 CM codes	n	%
**Infectious Diseases**				
	Human Immunodeficiency Virus (HIV)	042	38	0.8
**Endocrine, Nutritional And Metabolic Diseases, And Immunity Disorders**				
	Diabetes mellitus	250	512	10.8
**Mental Disorders**				
	Alcohol dependence syndrome	303	113	2.4
**Liver Diseases**				
	Chronic cirrhosis	571	132	2.8
	Alcoholic cirrhosis	571.2	14	0.3
	Non-alcoholic cirrhosis	571.5	9	0.2
	Biliary cirrhosis	571.6	1	0.02

### Spatial and temporal trends in Spain

**Fig 1:**
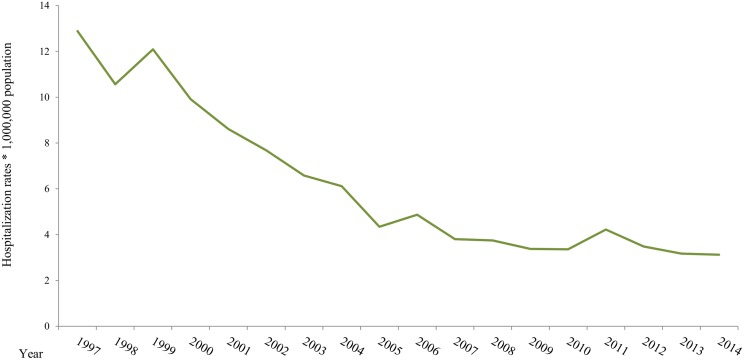
Mediterranean spotted fever hospitalizations rates by year, 1997–2014, Spain.

The median annual hospitalization rate was 5.6/1 million population (range 3.1–12.9/1 million population). The temporal trend of these hospitalizations is represented in Fig 1. From 1997 to 2014, a decreasing trend in the number of hospitalizations with MSF was observed. A steady decline from 1999 to 2005 was afterwards followed by a light increase in 2006, then a smoother decline till last year (2014).

**Fig 2:**
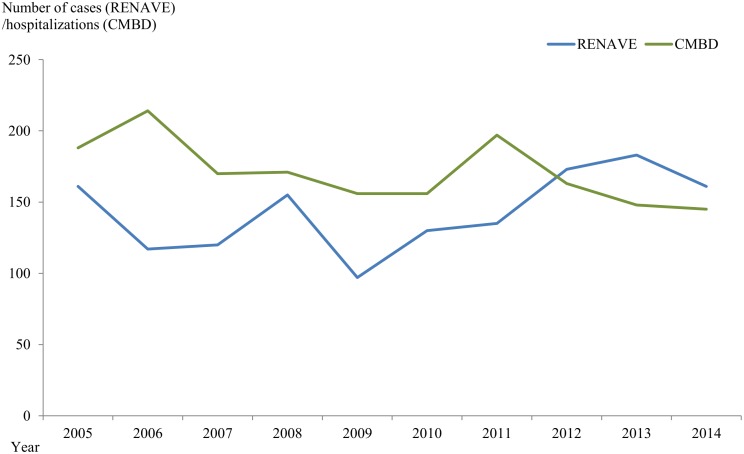
Comparison of the number of hospital registries (CMBD) of MSF patients (blue line) with those reported in the RENAVE (green line), from 2005 to 2014.

Comparison of the MSF hospital registries and the data officially reported in the RENAVE is shown in Fig 2 (from 2005 to 2014). Data from RENAVE was only available for this time period. The figure shows that the number of MSF cases reported in RENAVE was lower every year than the number of CE hospital discharges until 2012, when the difference reversed.

**Fig 3:**
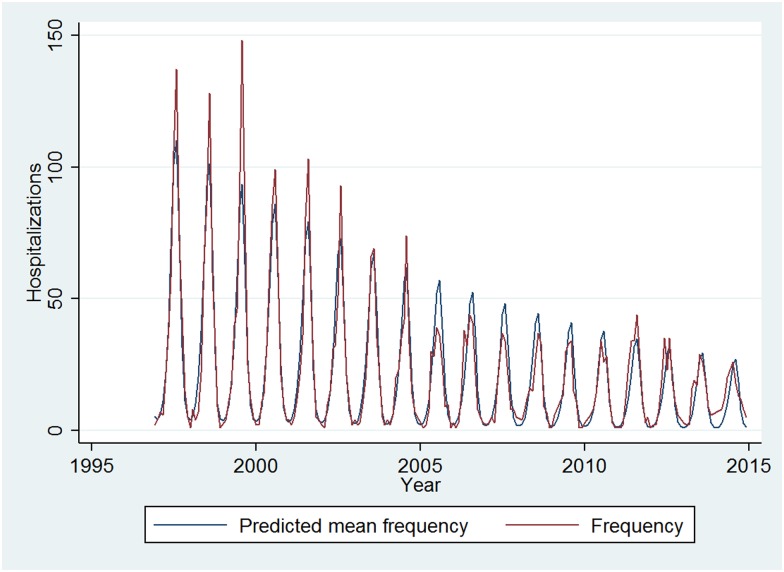
Mediterranean spotted fever hospital registries by month of admission, 1997–2014, Spain.

Most admissions occurred during the summer months, peaking in August. When analyzing the temporal behavior of the number of monthly hospitalizations, a significant and negative trend was found, indicating that the number of hospitalizations has been constantly decreasing in recent years. Regarding seasonality, both the terms corresponding to the annual cycle and those corresponding to the six-month cycle were significant, thus there is an annual seasonal behavior, producing the highest concentration of cases in the months of summer (Fig 3, S2 Table).

Regarding the regional distribution of MSF hospitalizations throughout the whole study period, all the autonomous communities registered discharges, even those considered as non-endemic. Huge differences were observed between autonomous communities (CC.AA). Ceuta had the highest median hospitalization rate (55.6/100,000 inhabitants), followed by Extremadura (25.9/100,000 inhabitants) and Rioja (25.2/100,000 inhabitants) (S1 Table).

**Fig 4:**
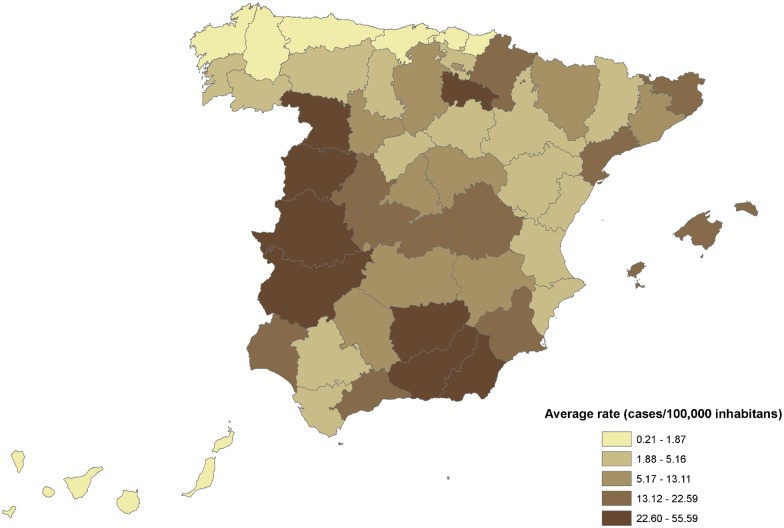
Hospitalizations rates (hospitalizations/100,000 inhabitants per year) by province, 1997–2014, Spain.

There were also significant differences between areas (Fig 4). The hospitalizations rates were above 25 admissions per 100,000 person-years in both Extremaduran provinces (Badajoz and Cáceres), three Andalusian provinces (Jaen, Granada y Almería), and two provinces of Castilla-Leon (Zamora and Salamanca). La Rioja and Ceuta (located in the north of Africa) showed the highest rate.

## Discussion

Our study indicates that most of the MSF hospitalizations were males aged over 45 years old. In most affected countries, rickettsioses cases commonly occurred among young males [20]. According to the CMBD database, MSF hospitalizations in Spain occurred more frequently in those above 45 years old. In a study carried out in Portugal, the highest incidence rate was observed in children (1–4 years old), though it was the elderly patients who had higher complication and mortality rates [21]. As the CMBD database included hospitalized patients (and not those treated at ambulatory level), we might have a selection bias for the most complicated forms of this disease.

MSF is usually mild but can have a severe course in patients with risk factors such as diabetes mellitus, heart failure, alcoholism, elderly or G6PD deficiency [21]. We checked in the CMBD database for other co-diagnosis previously described in the literature, in order to determine whether our hospital admissions were due to the increased presence of these factors among patients. No cases diagnosed as G6PD deficiency were found. Regarding the presence of diabetes mellitus, we found a prevalence of 10.8% among MSF hospitalizations, similar to the official prevalence data estimated by the International Diabetes Federation for Spanish adult population (10,4%) [22]. On the other hand, the presence of alcohol dependence syndrome (ADS) and HIV were slightly superior among the MSF hospital records than in overall Spanish population (2.4% vs. 1.5% and 0.8% vs. 0.4%, respectively), although official prevalence data for ADS and HIV varied considerably over the study period [23,24]. Finally, chronic liver disease and cirrhosis occurred in 2.8% of MSF hospitalizations. Unfortunately we did not find national morbidity data to compare with, only official data for associated mortality [25].

During the study period of 18 years, there were 4,735 hospital discharges for which MSF were recorded in any diagnostic position. In 4,375 records, MSF was placed in first diagnostic position. A global decreasing trend was observed in the Spanish territory, especially in the 1997–2005 period. In most Southern European countries, MSF appears to be waxing and waning. While incidence of the disease sharply increased in the 1980s in Italy, Spain, and southern France [26], peaking in the 1990s in Portugal [8] and Italy [26], it started decreasing in the mid-2000s [2]. On the other hand, in some countries, such as Bulgaria, MSF seems to be reemerging, after almost disappearing [27]. Such variations have also been described for Rocky Mounted spotted fever (RMSF) in United States [28]. These fluctuations in the incidence of a vector-borne disease such as the MSF are quite difficult to explain. Some possible explanations are changes in the epidemiologic surveillance of MSF cases; variations in the diagnostic tests that are used for the diagnosis of MSF; improvements in MSF control measures; or changes in human contact with the habitat of infected ticks, related, for example, with ecologic and climate changes. Improvements in the knowledge of the health care providers is another possible explanation, although to our knowledge there have been no national or regional campaigns targeting this issue in Spain in the last years.

In the last 20 years, the rickettsial field has undergone a substantial evolution thanks to the broad use of cell culture systems and the development of molecular methods [29]. Before, serologic techniques exhibited cross-reactivity between the spotted fever group (SFG) *Rickettsiae* thus, all rickettsioses with SFG antibodies were considered like MSF in countries or regions where this disease was endemic [2]. In the 18 years period of CMBD records that we have analyzed, we observed an increased in Q fever related hospitalizations, a constant decrease in MSF records and almost no trend changes in the rest of rickettsial related hospitalizations (S1 Fig). Probably, the improvements in MSF diagnostic methods already existed since the beginning of the study period.

The decrease in MSF hospitalizations may also be due to improvements in control measures. The risk of MSF can be reduced by tick control on dogs through periodic tick check and the use of pesticide and/or repellent products such as pyrethroids [30]. According to a survey carried out in the United States (US), pet owners are more concerned about fleas and ticks than any other parasites [31], especially after the US Environmental Protection Agency (EPA) issued several advisories reinforcing the importance of using spot-on flea and tick control products. We did not find further information for Europe, but the fact that Q persisted and even increased in the same time period is coherent with this hypothesis, as cattle, sheep, and goats are Q fever primary reservoirs and that Q fever infection results mainly from inhalation of a spore-like small-cell variant [32].

The comparison of RENAVE notified cases with hospital records performed here indicates discrepancy between both registries; there are more hospitalizations than notified cases until 2012, since when RENAVE data surpassed the CMBD records. To our knowledge, there has been no change in the MSF surveillance system in the last decade. On the other hand, we know that MSF is usually a mild disease [20, 33]; therefore most MSF cases do not require hospitalizations but yet should be officially notified. Thus, CMBD database should underestimate the real burden of MSF hospitalizations, what is not the case (at least not until 2012). Moreover, MSF is considered an endemic disease in few CCAA while emerging in others, though the case reporting is not mandatory in the whole country, only in those CC.AA. considered endemic [14]. Prospective data will help us in the future to enlighten this issue.

According to the CMBD database, admissions by MSF occurred mainly during the summer months. We know that in the Mediterranean basin most cases are encountered in late spring and summer [34]. The *Rhipicephalus sanguineus*, the vector for MSF in Europe, commonly called the brown dog tick, is active from spring to autumn, but climatic changes influence its activity and, consequently, the MSF epidemiology [1]. Increases in the incidence of MSF has even been associated with higher summer temperature, which in turn is related to the increased aggressiveness and propensity of *Rh*. *sanguineus* to bite hosts in warmer conditions [2].

Regarding the regional distribution, it varied among CC.AA and provinces. Overall, Extremadura had the highest mean hospitalization rate for the whole period, followed by Andalusia, Castilla-Leon, la Rioja and Ceuta. This geographical variability within the Spanish territory can be explained by the degree of diagnostic suspicion by health care workers or epidemiological factors, such as climate or vegetation [35]. Unfortunately, the epidemiology of rickettsiae and rickettsial diseases is not well discerned; we just know that Extremadura, Andalusia and Ceuta have warm to temperate climate, characterized above all by a pronounced summer drought, while Castilla-Leon and la Rioja have a mediterranean climate with dry warm summers and mild winters. Up to date, only partial research, targeting humans or vectors or dogs, have been carried out in some of these CC.AA. In the next future, the association between MSF and environmental factors like meteorological parameters and land use will be explored.

In Extremadura, a multicentric study was carried out in 1984; we are not aware of any more recent research on MSF in this CC.AA [36]. In Andalusia, some seroepidemiological studies have found a prevalence of past infection over 5% in healthy population, although these studies were carried out in Seville, which is one of the less MSF affected Andalusian provinces, according to our data [37,38]. In Castilla Leon, a seroepidemiological study was conducted in 308 dogs to determine the presence of antibodies to *Rickettsia conorii*. In this research, the provinces of Salamanca and Zamora showed the greater percentages of seropositive dogs [39], which is consistent with our results in humans. Special mention to La Rioja, where the Spanish Rickettsiosis and Arthropod-Vectors Borne Diseases Center is placed. In this CC.AA, even new spotted fever group *Rickettsia* have been described [40]. Nevertheless, we believe that a one health approach is missing in all these research, as we still find difficult to have a clear view of MSF human-host-vector spectra in the country. Besides, the lack of reliable statistics on the MSF epidemiology makes it difficult to compare prevalence rates before and after the application of control programs in Spain. Whatever the case may be, our results underline the need of a nationwide systematic approach towards CE.

## Limitations and conclusions

There are some aspects that need to be considered when interpreting the findings from this research. Our study only included cases of MSF requiring hospitalization, which is not equivalent to the true MSF incidence in the population. Even if the CMBD provide information from a network of hospitals that covers more than 99% of the population living in Spain [15], hospital discharge records do not include cases managed in an outpatient setting or asymptomatic cases, thus hospital records are still underestimating the real burden of MSF in Spain.

Another relevant limitation is that the CMBD does not provide information about laboratory test results for diagnosis of MSF, such as cell culture systems or molecular methods. However, the CMBD database provides reliable information to support decision-making based on ICD-9 codification carried out by medical doctors without being subject to the limitations of outpatient surveillance systems.

Data on the animal side of the MSF epidemiological scenario at national level are also missing. All these figures underline the need of a national surveillance system, which would facilitate a more accurate data collection, analyzing and interpretation. This will also result useful both in gaining extended disease knowledge and reducing morbidity and related-costs, but especially in evaluating implemented control actions.

## Supporting information

S1 Fig Hospitalizations coded as rickettsial diseases by year, 1997–2014, Spain. (TIF)

S1 Table Mediterranean botonous fever hospitalizations rates per 100,000 person-years by autonomous community, 1997–2014, Spain. (DOCX)

S2 Table Poisson regression analysis of monthly number of MSF hospitalizations, CMBD database, 1997–2014, Spain. (DOCX)
