# Supplementary figures and images for: Mediterranean spotted fever in Spain, 1997-2014: Epidemiological situation based on hospitalization records
Source: PLoS One. 2017 Mar 29;12(3):e0174745. doi: 10.1371/journal.pone.0174745 (PMC5371374; doi:10.1371/journal.pone.0174745)

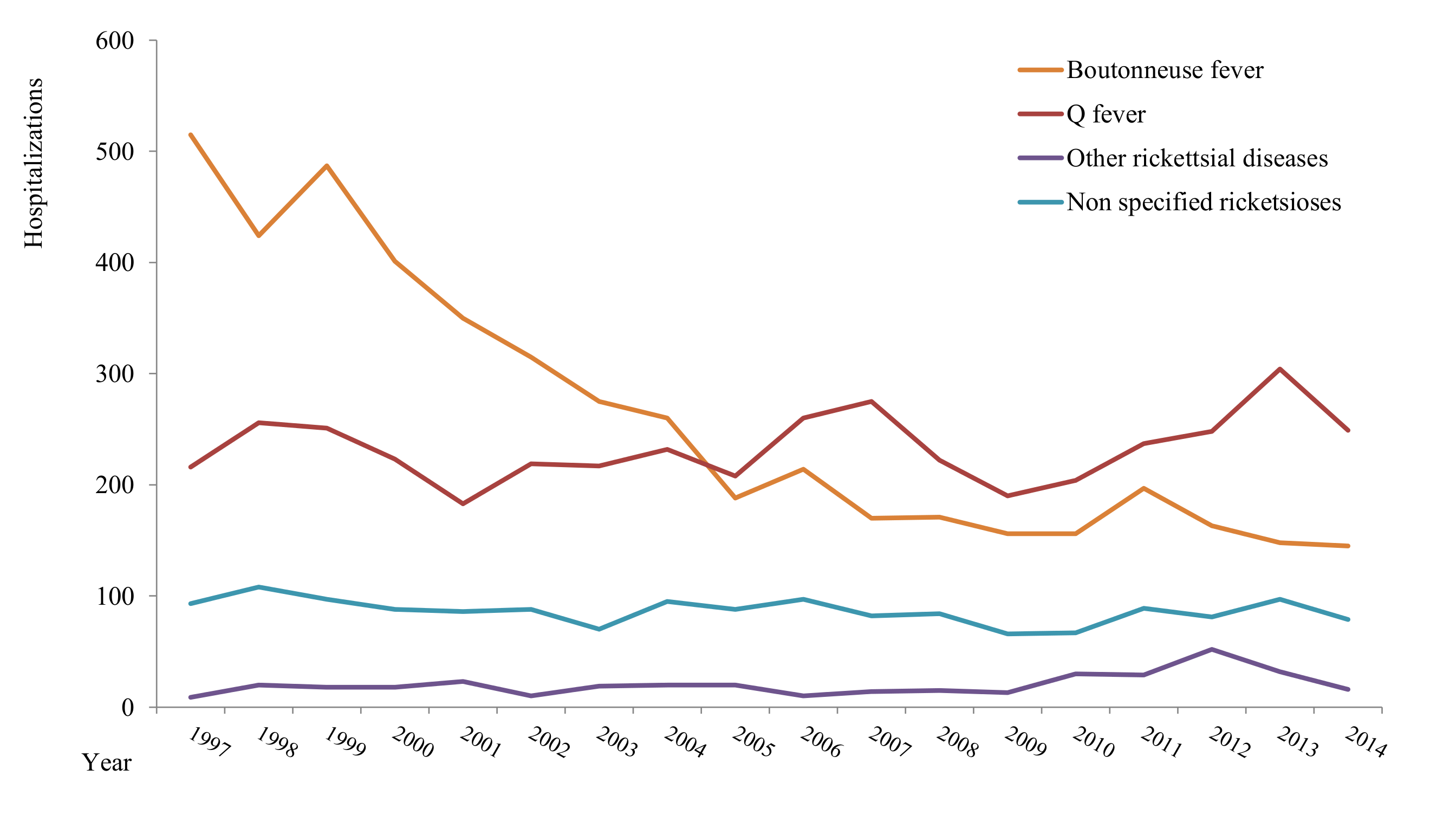

Supplement: S1 Fig — (TIF) [file pone.0174745.s001.tif]
