# Supplementary material for: Mediterranean spotted fever in Spain, 1997-2014: Epidemiological situation based on hospitalization records
Source: PLoS One. 2017 Mar 29;12(3):e0174745. doi: 10.1371/journal.pone.0174745 (PMC5371374; doi:10.1371/journal.pone.0174745)
Supplement: S1 Table — (DOCX) [file pone.0174745.s002.docx]

| **Supplementary Table 1. Mediterranean botonous fever hospitalizations rates per 100,000 person-years by autonomous community, 1997-2014, Spain.** | | | |
| --- | --- | --- | --- |
| **Autonomous community** | **Cases** | **Population average** | **Average rate** |
| **Andalusia** | 1,098 | 7,873,481 | 13.95 |
| **Aragon** | 51 | 1,271,451 | 4.01 |
| **Asturias** | 7 | 1,077,071 | 0.65 |
| **Balearic Islands** | 154 | 982,155 | 15.68 |
| **Canary Islands** | 5 | 1,934,239 | 0.26 |
| **Cantabria** | 3 | 563,194 | 0.53 |
| **Castilla-Leon** | 305 | 2,514,323 | 12.13 |
| **Castilla-La Mancha** | 261 | 1,918,806 | 13.60 |
| **Catalonia** | 877 | 6,949,231 | 12.62 |
| **Valencia** | 177 | 4,653,479 | 3.80 |
| **Extremadura** | 282 | 1,086,704 | 25.95 |
| **Galicia** | 65 | 2,758,216 | 2.36 |
| **Madrid** | 434 | 5,898,538 | 7.36 |
| **Murcia** | 245 | 1,323,399 | 18.51 |
| **Navarra** | 97 | 594,473 | 16.32 |
| **Basque Country** | 46 | 2,138,510 | 2.15 |
| **Rioja** | 75 | 297,877 | 25.18 |
| **Ceuta** | 43 | 77,356 | 55.59 |
| **Melilla** | 8 | 70,568 | 11.34 |
| **Total** | **4,233** | **43,983,069** | **9.62** |
