# Supplementary material for: Mediterranean spotted fever in Spain, 1997-2014: Epidemiological situation based on hospitalization records
Source: PLoS One. 2017 Mar 29;12(3):e0174745. doi: 10.1371/journal.pone.0174745 (PMC5371374; doi:10.1371/journal.pone.0174745)
Supplement: S2 Table — (DOCX) [file pone.0174745.s003.docx]

| Supplementary Table 2. Poisson regression anaysis of monthly number of MSF hospitalizations, CMBD database, 1997-2014, Spain. | | | | |
| --- | --- | --- | --- | --- |
|  | **Coef.** | **P>\|z\|** | **95% confidence interval** | |
| **Annual sine** | -1.5 | 0.0 | -1.6 | -1.4 |
| **Annual cosine** | -0.6 | 0.0 | -0.7 | -0.6 |
| **Biannual sine** | -0.2 | 0.0 | -0.3 | -0.1 |
| **Biannual cosine** | -0.1 | 0.0 | -0.1 | 0.0 |
| **Trend** | -0.01 | 0.0 | -0.01 | -0.01 |
| **Constant** | 6.2 | 0.0 | 6.0 | 6.5 |
